# Women and Treatment for Opioid Use Disorder: Contributors to Treatment Success From the Perspectives of Women in Recovery, Women With Past Attempts in Drug Treatment, and Health and Criminal Justice Professionals

**DOI:** 10.1177/11782218231222339

**Published:** 2024-01-10

**Authors:** Emma M Skogseth, Kristina Brant, Eric Harrison, Hannah B Apsley, Max Crowley, Robert P Schwartz, Abenaa A Jones

**Affiliations:** 1 Department of Human Development and Family Studies, The Pennsylvania State University, University Park, PA, USA; 2 Department of Agricultural Economics, Society, and Education, The Pennsylvania State University, University Park, PA, USA; 3 Friends Research Institute, Baltimore, MD, USA; 4 Consortium on Substance Use and Addiction, Penn State University, University Park, PA, USA

**Keywords:** Women, criminal justice, substance use, medications for opioid use disorder, treatment retention, criminal legal system

## Abstract

**Introduction:**

The disproportionate incidence of opioid use disorder (OUD) and the alarming increases in opioid-related overdose deaths among women highlight a clear need for the expansion of effective harm reduction and treatment practices. Research supports medications for opioid use disorders (MOUD) as an effective intervention; however, with low rates of utilization of such, there is a need to identify factors that facilitate MOUD treatment uptake and retention for women. Thus, the current study examines contributors to treatment success through the triangulation of perspectives from affected women as well as health and criminal justice professionals.

**Methods:**

Interviews (N = 42) were conducted from May to July 2022 with women in recovery who previously used or currently use MOUD (N = 10), women who currently use opioids who terminated a MOUD program previously (N = 10), SUD treatment professionals (N = 12), and criminal justice professionals who work with women who use opioids (N = 10). Interviews for all participants centered around their backgrounds, perceived barriers and facilitators to MOUD treatment, and issues specific to women in treatment for substance use disorder. We used a thematic qualitative data analysis process to analyze transcripts.

**Results:**

Participants highlighted contributors to treatment success from 3 domains: (1) internal processes (including promoting self-efficacy and setting realistic goals), (2) access to resources (including material resources, such as food and shelter, educational resources and social support), and (3) treatment structure (such as treatment type and protocol).

**Conclusion:**

Internal processes, access to resources, and treatment structure contribute to MOUD treatment success for women with OUD. Structured support where experiences are shared, and realistic goals are set, may promote feelings of acceptance and empowerment, thereby bolstering chances of treatment success. Additionally, the court system can promote evidence-based and trauma-informed substance use treatment and provide accessible educational resources related to substance use to extend these benefits to more women.

## Introduction

### Women and opioid use and overdose trends

The ongoing opioid crisis in the United States (U.S.) threatens the health and well-being of individuals, families, and communities. As of 2020, nationally representative data describe concerning trends among women; for people over the age of 11, women accounted for 55.6% of the estimated 2.7 million to have opioid use disorder (OUD), and 52.6% of the 9.5 million approximated to be misusing opioids—a departure from historic substance use patterns where use predominates among men over women.^1,2^ While opioid misuse affects people of all demographics, this differential impact of OUD on women demonstrates their importance as a population of focus.

Multiple reports discussing opioid use initiation points have found that women suffer from acute and chronic pain at greater rates than men, and to address this pain, women are prescribed opioids at a comparatively higher frequency and for a larger range of ailments.^3,4^ Additionally, many women with substance use disorders have experienced past trauma, and have symptoms of PTSD (Holzhauer et al) and other mental health concerns (Keyser-Marcus et al).^5,6^ Those without adequate access to health care may misuse prescription or illicit opioids to self-medicate, to cope with both physical pain as well as past trauma and mental health concerns.^7^ Research also suggests that it takes a shorter period for women to become opioid-dependent.^8^

Beyond looking at sex-based trends in opioid misuse, it is important to examine overdose rates. In 2021, more than 106 000 people in the United States died of a drug overdose, and 75.4% (80 411) of these deaths involved opioids (NSDUH).^9^ Non-methadone synthetic opioids, such as fentanyl, were involved in 87.8% (70 601) of all opioid-related deaths, followed by prescription opioids (20.8%; 16 706) and then heroin (11.4%; 9173).^9^ Women made up 31.4% of all drug overdose descendants in 2021, and 29.4% of overdose deaths related to opioid use, specifically.^9^ While men are dying in greater numbers, there has been an alarming increase in overdose deaths for women over the past 2 decades; overdoses among women have increased by over 500% since 1999, exceeding the increase of roughly 400% among men.^3,10,11^ This jarring increase in overdose deaths among women and male-female disparities in OUD make research focusing on women’s experiences with opioid use and treatment of the utmost importance.

### Opioid use treatment utilization in women

Given the disproportionate incidence of OUD and the alarming increase in opioid-related overdose deaths among women, there is a clear need to expand effective harm reduction and treatment practices. An impactful evidence-based method of OUD treatment is using medication for OUD (MOUD). MOUD treatment involves using methadone, extended-release naltrexone, or buprenorphine to disrupt cravings and suppress the euphoria association with the misuse of opioids.^12,13^ Several studies have highlighted the benefits of MOUD treatment, including lower rates of opioid misuse, reduced spread of blood-borne pathogens, decreased risk of overdose, and improved overall health and well-being.^14-18^ This evidence makes a strong case for the utility of MOUD in treating OUD and speaks to the need for greater accessibility to MOUD treatment.

While research supports MOUD as an effective intervention, there have been gaps in its delivery to individuals with OUD. It is important to note that estimating MOUD utilization is complicated by administrative record reliance, differing prescribing practices, and difficulty estimating how many adults have OUD.^19,20^ Despite such complexities, recent estimates paint a bleak picture of utilization rates: between 11% and 28% of people in the U.S. with OUD received MOUD in 2020.^2,21^ Additionally, present data lack estimates of MOUD utilization rates by sex. As women face unique experiences related to OUD and substance use treatment,^22^ it is imperative to examine their perspectives and those of substance use disorder (SUD) treatment and criminal justice professionals who work with women with OUD to understand further barriers to accessing MOUD.

### Barriers to initiating and retaining MOUD treatment

With most individuals with OUD not receiving MOUD, it is critical to look at obstacles to the initiation and retention in such treatment. Stigma is one of the most common reasons adults do not utilize MOUD or prematurely end treatment.^23^ Several studies on stigma indicate that there is great social and internal ambivalence tied to MOUD due to public perceptions of OUD as a choice, a prevailing view that MOUD is “just another drug,” and social isolation from friends, coworkers, and family members alike that contributes to the internalization of negative.^24-26^ Notably, research also shows that stigma disproportionately impacts women’s treatment initiation and retention.^27-29^ Stigma is a powerful obstacle to initiating and continuing MOUD use and is worthy of future study incorporating multiple perspectives.

In addition to stigma, several other factors that obstruct treatment uptake and retention stem from general inaccessibility. These include insurance coverage that excludes certain MOUD costs, a lack of transportation to clinic appointments, limitations on take-home dosages, and shortages of providers that contribute to long waiting lists and further distances between individuals and treatment centers.^23,30,31^ Moreover, several additional barriers are specific to women, including fears of losing custody of one’s children, some providers’ unwillingness to provide methadone or buprenorphine treatment to pregnant women, and difficulties finding childcare during treatment appointments.^8,22,28,32^ These accessibility-related and gender-specific barriers impede the ability of women with OUD to initiate and continue treatment.

A subgroup of women likely disproportionately affected by these barriers to MOUD uptake and retention are those with a history of involvement in the criminal justice system. Justice-involved women tend to have low rates of employment, lack transportation, have no insurance or limited coverage, and experience a greater likelihood of housing instability.^33-35^ These are all barriers related to accessibility that can further complicate seeking and staying in treatment often beyond that of women in the general population, which emphasizes the importance of research focusing on identifying what *is* benefiting their access to and continued use of treatment. Ultimately, while there is a sizable body of literature examining barriers to MOUD use and drawing on the viewpoints of singular groups, greater attention should be afforded to factors that facilitate MOUD uptake and retention, especially through the triangulation of the accounts of both individuals with OUD and the SUD treatment professionals who serve them; thus, with a focus on justice-involved women, the present study aims to fill this gap.

### Aim of study

Previous studies examine barriers to MOUD utilization; however, few focus on the factors which facilitate, rather than impede, MOUD treatment success. Additionally, few draw specifically on insights from women and the individuals in their nested social contexts. Given this, the present study aims to identify factors contributing to successful MOUD treatment among women with OUD using the perspectives of affected women as well as those of SUD treatment and criminal justice professionals. We focus specifically on factors contributing to *retention* in MOUD treatment. This work is vital, as it can shed light on those factors contributing to MOUD treatment success; incorporating such elements into the development of practice and policy could enable more women to access and benefit from MOUD treatment, sustaining positive outcomes long-term, especially a drastically reduced likelihood of fatal overdose.^36,37^

## Methods

We used semi-structured qualitative interviews (N = 42) from May to July 2022 to gain in-depth insights regarding contributors to MOUD treatment success and retention from multiple perspectives. Participants included women in recovery who previously used or currently use medications for opioid use disorder (MOUD) (N = 10), women who currently use opioids who terminated a MOUD program previously (N = 10), SUD treatment professionals (N = 12), and criminal justice professionals who work with women who use opioids (N = 10). In this context, the use of the term “opioids” is broad, as some women used only prescription opioids, some used only illicit opioids, and some used a combination of both. Additionally, treatment “success” was defined by one’s provider as the participant completing the MOUD treatment program or continuing long-term maintenance treatments with MOUD. This meant that they were not illicitly using opioids; many were continuing to take medications, attend therapy, and engage in other harm reduction practices. See Table 1 for a list of the roles held by the SUD treatment and criminal justice professionals. Penn State’s Institutional Review Board approved this study.

**Table 1.** Roles of criminal justice and SUD treatment professionals.

Criminal justice professionals		SUD treatment professionals	
*Role*	*N*	*Role*	*N*
Prosecutors	2	MOUD provider	1
Law enforcement	3	Counselor	3
Treatment Court professional	4	Recovery/treatment program director	2
Corrections officer	1	Research assistant	1
		Case manager/recovery coach	3
		Nurse	2

### Participant recruitment

The recruitment and eligibility criteria were tailored to the group in question: the women with a history of taking MOUD, the SUD treatment professionals, and the criminal justice professionals who work with women who use opioids. To recruit the 20 women, we used a targeted sampling strategy involving social media advertisements and chain referrals. We also sent flyers to MOUD treatment programs in Pennsylvania (PA.) to advertise the study. To be eligible for the interview, the women had to provide consent; be 18 years or older; have a lifetime history of being on probation, parole, or incarcerated; reside in PA.; and have a lifetime history of MOUD program enrollment with 10 participants defined by their healthcare professionals as having achieved recovery through MOUD treatment and the other half having previously terminated from such treatment. Notably, some of the programs that participants had participated in also included other types of treatment, such as therapy or mutual help groups, but all participants had been prescribed MOUD as at least part of their treatment regimen at one point in time, or were still currently taking MOUD.

The 12 SUD treatment professionals were recruited from a directory of all 103 Opioid Treatment Programs (OTPs) licensed by the Pennsylvania Department of Drug and Alcohol Programs by direct calls and chain referrals. To participate in the interview, the SUD treatment professionals had to be healthcare workers who prescribed MOUD, cared for those being treated with such, and/or provided counseling as a component of a MOUD treatment program. Further eligibility also included that they must practice in PA, work with women clients with OUD, and consent to the interview.

Similar to reaching the SUD treatment professionals, recruiting the 10 criminal justice professionals involved placing direct calls to women’s prisons in PA, chain referrals, and online advertisements. To be eligible for the interview, the criminal justice professionals had to provide informed consent, practice in PA, and hold one of the following positions: parole or probation officer, judge, or drug court professional. Eligibility criteria also held that their work had to be with women involved in the criminal justice system probation and using substances.

A brief web screening was provided to ensure all those interested met the eligibility requirements. The screening involved a REDCap survey of information regarding age, sex, other demographic items, and contact information. For individuals who did not meet the criteria for eligibility, this survey automatically concluded, and they were screened out.

### Interviews

Following recruitment, trained study staff called eligible participants to schedule interviews. Each interview was an hour long on the scheduled day and took place over the phone. The interview consisted of 13 questions that focused on either their experiences with MOUD treatment or work with women who use drugs, as well as the insights gleaned from such experiences. In this context, when using the term “MOUD treatment,” the women are referring to different types of treatment settings, such as inpatient, outpatient, and medication-assisted treatment clinics, and are discussing their successes and challenges with MOUD across these settings. For their time, participants were given a $50 gift card as compensation.

Every call began with the staff going through the informed consent process with the potential participant. If the participant gave their verbal consent, then the interview started. Additionally, interview staff clarified that participants were free to decline to answer any questions they were uncomfortable with or did not wish to answer.

### Data analysis

Trained study staff conducted, transcribed, and stored interview data on secure servers, using the software *NVivo*. To protect confidentiality, data was de-identified using pseudonyms for participants. Three members of the research team then carefully read transcripts to initially come up with an inductive list of primary codes. To continue generating these primary codes, the 3 team members kept closely reviewing transcripts until no additional primary codes were required to be included in the codebook from the content of new transcripts. Then, within each primary code, the 3 team members repeated this process for the generation of secondary codes. Given that interviewers asked participants of all sub-sample groups similarly structured questions to discern points of divergence and convergence between affected women and the criminal justice and SUD treatment professionals who serve them, the research team generated a single codebook for utilization in all groups. A different group of 4 members of the research team, PI included, used this codebook to code every transcript with one team member coding each transcript. For consistency, the PI double checked all coded transcripts. This paper utilizes the primary code “Facilitators to Treatment Success.”

## Results

### Participant characteristics

Overall, 42 participants were recruited: 10 women in recovery who previously used or currently use MOUD, 10 women who previously terminated a MOUD program and currently use opioids, 12 SUD treatment professionals, and 10 criminal justice professionals. The 20 women with a lifetime history of MOUD use ranged in age from 24 to 54 (mean 37 years), and most identified as white, non-Hispanic (n = 14, 70%). Of the SUD treatment professionals, 10 identified as female, and the remaining were male. Their ages ranged from 38 to 54 (mean 48 years), and the majority identified as white, non-Hispanic (n = 8, 66.7%). Seven criminal justice professionals identified as female; the remaining were male. One criminal justice professional identified as Black, and 9 identified as white, non-Hispanic. Their average age was 44 years, ranging from 34 to 56. See Table 2 for participant demographics by subgroup.

**Table 2.** Sociodemographic characteristics by group.

Women with OUD	n (%)	M (SD)
Age		37 (7)
Sex		
Women	20 (100%)	
Race		
White	14 (70%)	
Black	1 (5%)	
Biracial	1 (5%)	
N/A	4 (20%)	
Education		
Highschool	13 (65%)	
College	6 (30%)	
N/A	1 (5%)	
Children		
Yes	14 (70%)	
No	6 (30%)	
MOUD treatment		
Completed	5 (25%)	
Left AMA	3 (15%)	
Noncompliance	2 (10%)	
Disruption	4 (20%)	
N/A		
SUD treatment professionals	n (%)	M (SD)
Age		48 (5)
Sex		
Male	2 (17%)	
Female	10 (83%)	
Race		
White	8 (66%)	
Black	2 (17%)	
N/A	2 (17%)	
Education		
Highschool	2 (17%)	
College	4 (33%)	
Advanced degree	3 (25%)	
N/A	3 (25%)	
Criminal justice professionals	n (%)	M (SD)
Age		44 (8)
Sex		
Male	3 (30%)	
Female	7 (70%)	
Race		
White	9 (90%)	
Black	1 (10%)	
Education		
Highschool	1 (10%)	
College	1 (10%)	
Advanced degree	7 (70%)	
N/A	1 (10%)	

### Themes

**Figure 1.**
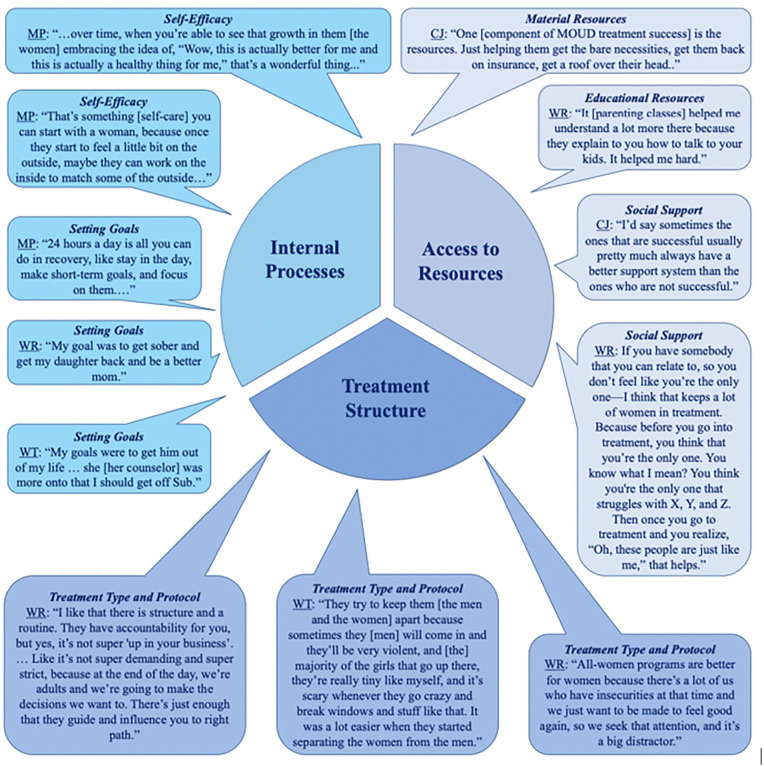
Visualization of participants’ responses by domain.

To identify factors contributing to successful MOUD treatment, we conducted qualitative telephone interviews with affected women, healthcare workers, and criminal justice professionals. We specifically asked each of these groups of participants about the factors they believe contribute to MOUD treatment success. A thematic analysis revealed that participants’ responses spanned 3 domains of contributors to MOUD success: internal processes, access to resources, and treatment structure. See Figure 1 for a visualization of participants’ responses.

### Internal processes

Internal processes are a distinct category involving women’s beliefs, emotional experiences, and individual commitments with OUD. Specifically, responses in this domain described women’s sense of self-efficacy and the setting of realistic goals for themselves. Delving into each of these sub-topics aids in painting a well-rounded picture of how internal psychological and behavioral processes can contribute to MOUD treatment success.

#### Self-efficacy

From the perspectives of SUD treatment professionals, feelings of self-efficacy emerged as a unique internal psychological process that contributed to treatment success. SUD treatment professionals described the value of bolstering patients’ feelings of self-efficacy by building their competencies and self-image while receiving MOUD treatment. Feelings of self-efficacy also often included belief in a higher power or the self.

Many participants linked self-efficacy to self-care; when women with OUD invested time in caring for their well-being, SUD treatment professionals described positive changes in feelings of self-efficacy. Speaking of one current successful client in particular, one SUD treatment professional, a treatment program director, delved into this in further detail:

That’s something [self-care] you can start with a woman, because once they start to feel a little bit on the outside, maybe they can work on the inside to match some of the outside. . . . A lot of times it’s the opposite. They just work on the outside and not the inside. Those little steps, like just the self-care, have been effective with this individual.

Within this response, the treatment program director touched on how improving one’s outside appearance—such as building self-care routines—could lead women to also do “inside work”—such as bolstering one’s sense of self and self-esteem—allowing women to thrive in treatment. Speaking to this further, a research assistant touched on a similar observed transformation of self-efficacy through women’s involvement with OUD treatment generally:

They [the women] arrive to you and they’re very bitter and resentful and angry that this is the situation and really don’t want to be there and are pretty clear about that, but then as you’re working with them and, over time, when you’re able to see that growth in them embracing the idea of, “Wow, this is actually better for me and this is actually a healthy thing for me,” that’s a wonderful thing to observe.

Through their answer, this SUD treatment professional touched on how they see noticeable growth and empowerment within those individuals who succeed in treatment. In their view, women who succeed have a moment of realization that treatment can be positive for them, and they are taking steps to build a healthier life for themselves. Ultimately, SUD treatment professionals highlighted that self-efficacy did not only contribute to women’s retention in MOUD-involved treatment, but that progressing in their treatment plan helped these women, in turn, to build self-efficacy.

#### Setting goals

Engaging in goal setting stands out alongside building self-efficacy as a theme within the domain of internal processes. SUD treatment professionals explained that MOUD treatment programs often seek to facilitate the development of reasonable action goals and the recognition of what may not be realistic. Women who succeeded in setting realistic goals often were likely to succeed in treatment overall; however, what these goals entailed seemed to differ between SUD treatment professionals and the women.

To SUD treatment professionals, reaching reasonable “success” metrics often involves breaking progress into smaller steps. Speaking to this in greater depth, one SUD treatment professional, a treatment program director, explained:

Any kind of progress is any kind of baby steps in a right direction. Out of this level, going to the next level, like a halfway house or three-quarter way house, or just even if they return home, 24 hours a day is all you can do in recovery, like stay in the day, make short-term goals and focus on them, not expecting too much out of yourself. That’s enough for us.

The treatment program director’s response framed setting realistic goals as progressing on a day-by-day timeline. The sentiment of day-to-day progress seems to be further exemplified by the provider defining success as completing a day without using drugs.

Answers from women with OUD were different in that they seemed to describe realistic goal setting as pursuing aspirations that were of the greatest importance to them. When asked about her goals during treatment, one participant in recovery stated that “my goal was to get sober and get my daughter back and be a better mom.” A different participant who had taken part in but then left a MOUD program also set her goal on what was most relevant to her needs, saying, “the goal was to get away. I had married someone after 6 weeks of knowing him. My goals were to get him out of my life and his name off of everything that I owned without losing it.” When asked if her goals differed from her treatment center’s, she told us: “Yes. She [her counselor] was more onto, ‘I should get off Sub [suboxone – buprenorphine]. Yes, I need to get rid of Fyl [fentanyl]’.”

Overall, participants highlighted setting goals as a contributor to MOUD treatment success; however, what SUD treatment professionals viewed as most important seemed to diverge from what the women identified as driving forces for treatment retention.

### Access to resources

Women, SUD treatment professionals, and criminal justice professionals also discussed the importance of resource access. While MOUD treatment focuses on providing participants with medications, success hinges on connecting women with other resource needs. Resource access is distinct from internal processes in that it includes specific factors *outside* the individual contributing to treatment success. The analysis of participants’ responses in this domain revealed material and social support, in particular, as prominent subtopics. Examining these emergent themes in greater depth contributes to understanding the role of resource access in bolstering the retention of MOUD treatment.

#### Material and educational resources

In this study, accessing educational and material support was described as women with OUD gaining knowledge about available social services like Medicaid and participating in educational programs like parenting classes. SUD treatment professionals, criminal justice professionals, and women alike discussed how treatment success hinged on women simply being able to meet their basic needs. One criminal justice professional, a mental health court coordinator, noted:

One [component of MOUD treatment success] is the resources. Just helping them get the bare necessities, get them back on insurance, get a roof over their head. The basic necessities of life, helping them do that stuff first. Just get them stabilized, hooked up with treatment quickly instead of waiting for months for an appointment.

Regarding material support, this explanation from the criminal justice professional covered how food, shelter, and resources necessary for safety were important components bolstering treatment success.

Beyond material resources, other components of this category named in participant responses were educational classes and resources regarding handling difficult situations, like navigating romantic relationships and parenting. Speaking to the latter, one participant in recovery stated that “it [parenting classes] helped me understand a lot more there because they explain to you how to talk to your kids. It helped me hard.” The participant told us how important it was to learn to speak to her daughter about her OUD to focus on her treatment and recovery. When we asked her what treatment should provide participants, she told us: “Anything to help with that [parenting], because I didn’t know how to explain it [her addiction] to her [her daughter].” Overall, many participants emphasized opportunities to gain educational and material resources as impactful contributors to MOUD treatment success.

#### Social support

Social support was a second theme of resource access that stood apart from gaining educational and material resources to contribute to treatment success. Separate from social services, social support was defined as having peer mentors with lived recovery experience to meet with while seeking treatment, as well as including family and friends in treatment. An example of the former includes the sense of community brought on by realizing that one is not alone. One participant who had successfully achieved recovery using MOUD touched on this:

If you have somebody that you can relate to, so you don’t feel like you’re the only one—I think that keeps a lot of women in treatment. Because before you go into treatment, you think that you’re the only one. You know what I mean? You think you’re the only one that struggles with X, Y, and Z. Then once you go to treatment and you realize, “Oh, these people are just like me,” that helps.

Her answer to the question of what factors aid in treatment retention highlighted the invaluable role of acceptance and being able to relate to others without judgment. Other women with OUD expressed similar feelings of understanding as being beneficial to their MOUD treatment retention in their responses, as did SUD treatment professionals.

Another component of social support was a general emotional support system. Whether it included family members, friends, a support group, or a counselor, the importance of having an emotional support system was a recurring answer given by women with OUD, SUD treatment professionals, and criminal justice professionals under this subtopic. One criminal justice professional, a treatment court coordinator, stated:

I’d say sometimes the ones that are successful usually pretty much always have a better support system than the ones who are not successful. It’s pretty much with anybody, but especially with females, if they don’t have a support system in place and not just being family, but any support system, they usually will not be successful.

This response concluded that having a support system was said to play an important role in whether MOUD treatment was successful. In this regard, it echoed the conclusions of similar answers given by other participants under this subtopic; feeling supported by others while seeking treatment was identified as a contributing factor to MOUD treatment retention by women with OUD.

### Treatment structure

The third domain highlighted by participants as contributing to MOUD treatment retention was treatment structure. This topic differs from internal processes and access to resources with its narrower scope: the focus is exclusively on elements of MOUD treatment programs that give them structure, such as consistency, routines, and mechanisms for accountability. Examining responses from the study’s participants in this domain identified treatment structure and type as a pertinent subtopic to further explore identifying factors that contribute to successful MOUD treatment.

#### Treatment type and protocol

The emergent theme from the domain of treatment structures captured by participant responses was the type and structure of treatment. The coding scheme described this as a reference to different styles of treatment and how they support retention. The SUD treatment professionals and women who had gone through MOUD treatment programs spoke to this further, as a recurring answer under this subtopic was the role of striking the right balance between structure and flexibility in treatment program protocol in bolstering successful outcomes. One participant who had successfully achieved recovery through a MOUD program described this further:

I like that there is structure and a routine. They have accountability for you, but yes, it’s not super “up in your business.” You know what I mean? Like it’s not super demanding and super strict, because at the end of the day, we’re adults and we’re going to make the decisions we want to. There’s just enough that they guide and influence you to right path.

This response to the question of the most helpful component of her treatment program demonstrates the benefit of programs providing structure and accountability while recognizing the autonomy of those in it.

In addition to balancing structure and flexibility, another beneficial element of treatment framework that came up in participant responses was the practice of separating men from women. The same participant as above stated that “all-women programs are better for women because there’s a lot of us who have insecurities at that time and we just want to be made to feel good again, so we seek that attention, and it’s a big distractor.” A different participant who took part in but then left an MOUD program concurred:

They try to keep them [the men and the women] apart because sometimes they [men] will come in and they’ll be very violent, and [the] majority of the girls that go up there, they’re really tiny like myself, and it’s scary whenever they go crazy and break windows and stuff like that. It was a lot easier when they started separating the women from the men. Also, because the men would always be like, “Hey, yo, I got a hookup, you should come with me.” Every girl would be like, “Come on, leave me alone. I’m trying to get better.”

Both participants had similar answers to the question of what helps women stay in MOUD treatment regarding separating men from women to minimize distractions and threats of violence. For the category of treatment structures overall, responses from SUD treatment professionals and women with OUD touched on helpful treatment programs, such as protocols that balanced structure with flexibility and all-women programs. Altogether, these elements of treatment structures were illustrated by participant responses as factors contributing to successful MOUD treatment outcomes.

## Discussion

### Findings

This study identified contributors to MOUD treatment retention and success through in-depth qualitative interviews from multiple perspectives. Emergent themes encompassed internal processes, resource access, and treatment structure. Overall, results revealed that the affected women, SUD treatment professionals, and criminal justice professionals’ perspectives converged on recognizing the importance of resources and treatment structure; however, SUD treatment professionals seemed to attribute more to internal processes while the women emphasized structure as most valuable to MOUD treatment retention.

The points of convergence across interview responses involved access to resources and treatment structure. Within these external factors, one prevalent topic identified by all participant groups from the interviews was education. Past studies focused mainly on educational programs during MOUD treatment about drug use and overdose.^28,38,39^ While also touching on how gaining this information contributed to MOUD treatment retention, the current study expanded previous work as participants discussed program-based education, including everything from parenting classes to social service eligibility and processes for obtaining resources. These findings suggest that incorporating multifaceted educational experiences into MOUD programs may be a valuable avenue to pursue when creating effective treatment interventions.

In addition to access to educational programing, participants’ discussions on the importance of social support in contributing to MOUD treatment retention and completion aligned with the conclusions of previous research.^27,39,40^ Some participants touched on how social support contributes to feelings of acceptance and understanding, which prior studies identified as highly valuable to combatting stigma, supporting emotional well-being, and contributing to recovery overall.^28,41-43^ Ultimately, a support system appears to be a major factor in treatment success, and future interventions should strive to maximize this to bolster effectiveness.

Shifting to the theme of treatment structure, one notable finding discussed by participants was how women-specific substance use treatment supports retention and successful outcomes. This conclusion corroborated with results from a study on veteran women with OUD and findings on women in drug court.^44,45^ Separating men from women in treatment can aid overall success by minimizing distractions and other risk factors, and this is worthy of further consideration when developing effective MOUD treatment interventions specific to the needs of women with OUD.

While participants’ perspectives appeared to converge on the value of treatment structure and resource access, there were some areas of divergence. Specifically, SUD treatment professionals seemed to attribute more successful outcomes to internal processes. One notable finding in this domain articulated by SUD treatment professionals was the role of self-efficacy in facilitating successful outcomes. Their discussions on the importance of the individual feeling competent in their ability to do the “inside work” in tandem with participating in the external treatment aligned with previous research highlighting individuals’ self-efficacy as a protective factor for successful MOUD treatment outcomes.^46,47^ Although self-efficacy was a prominent theme in SUD treatment professionals’ responses and past studies, it is worth noting that it was not touched on by the responses from the women with a lifetime history of MOUD treatment.

Alongside findings pertaining to self-efficacy, the internal process of setting goals was another discord between SUD treatment professionals and affected women. In concordance with past literature, both groups of participants spoke about the value of setting^46,48^; however, the goals they defined as beneficial to MOUD treatment retention differed. SUD treatment professionals spoke to the importance of breaking long-term aspirations into smaller, more attainable steps, like taking sobriety day by day. In contrast, the women tended to frame the benefits of goal setting as pursuing aspirations that were most valuable to them, such as becoming a better mom.

Overall, SUD treatment professionals talked more about the value of internal processes, whereas the women often spoke more about treatment structure. While both groups highlighted setting goals as contributing to successful treatment outcomes, they diverged in what those goals entailed. These findings may indicate a greater disconnect between what SUD treatment professionals view as important versus what factors women view as being most impactful. As women with OUD are those intended to benefit from these MOUD programs, future interventions should focus on the structure around women instead of centering work around internal processes.

### Limitations

This study has limitations worthy of further discussion. First, it is important to consider the role of self-report. Results may have been impacted by social desirability bias as individuals from all groups may have wanted to present their experiences more positively or to sound more favorable to the interviewer. Alongside participant confidentiality, the modality of the telephone interview may have mitigated this, as not being face-to-face helps ensure anonymity.

Additionally, only one of the providers recruited for this study was a medical physician able to prescribe MOUD themselves. The other providers who were interviewed worked with women receiving MOUD, but were not qualified to prescribe MOUD themselves (such as nurses and counselors). Future work on MOUD utilization could be strengthened by assessing the experiences of more prescribers; however, perspectives from counselors and nurses also strengthen the study in that these SUD treatment professionals work more closely with the women and could speak more in-depth about their lives and struggles.

The aim of this study was to assess facilitators to treatment success for women, as such, we cannot compare perspective shared by gender. Moreover, this study was not designed to represent all women with OUD or their SUD treatment and criminal justice professionals as it targeted the perspectives of those in PA recruited for this particular qualitative study. Nonetheless, by focusing on women with a lifetime history of MOUD treatment, the insights from this study are an important first step in recognizing and gaining greater understanding of potentially gender-specific factors influencing MOUD treatment uptake and retention among women. Future research can utilize this study’s findings to investigate whether similarities and differences exist when comparing experiences of such facilitators across individuals of different gender identities.

Finally, the subjectivity of defined terms such as MOUD treatment “success” and “structure” emerged as limitations to this study. Regarding “success,” we acknowledge that our definition is quite narrow in comparison to the broader, multi-dimensional, holistic outcomes described by participants in our study and other current work.^49^ Moreover, “structure” may also be a limited definition, as different programs often follow different procedures. Despite the subjectivity, insight from the participants on “success” and “structure” offers great value to the study through discussions of contributors to treatment retention and completion. Future work could utilize more holistic definitions of both terms to see if participant perspectives continue to touch on these points or identify further contributors.

## Conclusions

In conclusion, the direct experiences of justice-involved women with a lifetime history of MOUD treatment as well as those of SUD treatment and criminal justice professionals are powerful as they provide invaluable insight and can inform the development of more impactful interventions for women with OUD. To best serve women with OUD, the results from our study suggest that interventions should focus on the structure around women—their social support, material and educational resources, and treatment protocol—over internal processes. Findings from this study hold important implications for this area of research in that they can help in developing more effective, relevant MOUD treatment programs; direct funding, resources, and research toward evidenced contributing factors; and shape evidence-based, trauma-informed policy and practice. Noting the criminal justice focus of the sample, these results can also be valuable to the development of interventions, policies, and practices specific to justice-involved women, for the present study shed light on contributors to MOUD treatment retention and completion that were effective for this subgroup in particular. Future work triangulating these perspectives in other communities can strengthen the knowledge base around factors that facilitate MOUD retention and build a foundation upon which effective treatment programs can be developed.

## Supplemental Material

sj-docx-1-sat-10.1177_11782218231222339 – Supplemental material for Women and Treatment for Opioid Use Disorder: Contributors to Treatment Success From the Perspectives of Women in Recovery, Women With Past Attempts in Drug Treatment, and Health and Criminal Justice Professionals Supplemental material, sj-docx-1-sat-10.1177_11782218231222339 for Women and Treatment for Opioid Use Disorder: Contributors to Treatment Success From the Perspectives of Women in Recovery, Women With Past Attempts in Drug Treatment, and Health and Criminal Justice Professionals by Emma M Skogseth, Kristina Brant, Eric Harrison, Hannah B Apsley, Max Crowley, Robert P Schwartz and Abenaa A Jones in Substance Abuse: Research and Treatment

## References

[1] Centers for Disease Control and Prevention. National drug overdose (O.D.) Deaths, 1999-2021. CDC WONDER. 2023. Accessed April 26, 2023. http://wonder.cdc.gov/mcd-icd10.html

[2] Substance Abuse and Mental Health Services Administration. Key substance use and mental health indicators in the United States: Results from the 2020 National Survey on Drug Use and Health (Publication No. PEP21-07-01-003, NSDUH Series H-56). Center for Behavioral Health Statistics and Quality, Substance Abuse and Mental Health Services Administration; 2021. Accessed April 26, 2023. https://www.samhsa.gov/data/sites/default/files/reports/rpt35325/NSDUHFFRPDFWHTMLFiles2020/2020NSDUHFFR1PDFW102121.pdf

[3] Goetz TG Becker JB Mazure CM. Women, opioid use and addiction. FASEB J. 2021;35:e21303. 10.1096/fj.202002125R 33433026

[4] Zelaya CE Dahlhamer JM Lucas JW Connor EM. Chronic pain and high-impact chronic pain among U.S. adults, 2019. NCHS. 2020;390:1-8. 33151145

[5] Keyser-Marcus L Alvanzo A Rieckmann T , et al. Trauma, gender, and mental health symptoms in individuals with substance use disorders. J Interpers Violence. 2015;30:3-24. 24811286 10.1177/0886260514532523 PMC4766974

[6] Holzhauer CG Cucciare M Epstein EE. Sex and gender effects in recovery from alcohol use disorder. Alcohol Res Health. 2020;40:03-19. 10.35946/arcr.v40.3.03 PMC7668196 33224697

[7] McHugh RK DeVito EE Dodd D , et al. Gender differences in a clinical trial for prescription opioid dependence. J Subst Abuse Treat. 2013;45:38-43. 23313145 10.1016/j.jsat.2012.12.007 PMC3626739

[8] Barbosa-Leiker C Campbell ANC McHugh RK Guille C Greenfield SF. Opioid use disorder in women and the implications for treatment. Psychiatr Res Clin Pr. 2021;3:3-11. 10.1176/appi.prcp.20190051 PMC8639162 34870109

[9] National Institute on Drug Abuse. Drug Overdose Death Rates. National Institute on Drug Abuse; 2023. Accessed October 30, 2023. https://nida.nih.gov/research-topics/trends-statistics/overdose-death-rates

[10] Hedegaard H Miniño AM Spencer MR Warner M . Drug overdose deaths in the United States, 1999–2020. NCHS. 2021:1-8. https://stacks.cdc.gov/view/cdc/112340 34978529

[11] Ho JY. Cycles of gender convergence and divergence in drug overdose mortality. Popul Dev Rev. 2020;46:443-470. 33583972 10.1111/padr.12336 PMC7880043

[12] Shulman M Wai JM Nunes EV. Buprenorphine treatment for opioid use disorder: an overview. CNS Drugs. 2019;33:567-580. 31062259 10.1007/s40263-019-00637-z PMC6585403

[13] Volkow ND Jones EB Einstein EB Wargo EM. Prevention and treatment of opioid misuse and addiction: a review. Arch Gen Psychiatry. 2019;76:208-216. 10.1001/jamapsychiatry.2018.3126 30516809

[14] Bell J Strang J. Medication treatment of opioid use disorder. Biol Psychiatry. 2020;87:82-88. 31420089 10.1016/j.biopsych.2019.06.020

[bibr15-11782218231222339] Santo T Clark B Hickman M , et al. Association of opioid agonist treatment with all-cause mortality and specific causes of death among people with opioid dependence: a systematic review and meta-analysis. JAMA Psychiatr. 2021;78:979-993. 10.1001/jamapsychiatry.2021.0976 PMC8173472 34076676

[bibr16-11782218231222339] Springer SA. Hepatitis C virus reinfection rate among persons who use drugs and are maintained on medication treatment for opioid use disorder. Clin Infect Dis. 2020;70:2703-2705. 31346595 10.1093/cid/ciz695 PMC7286379

[bibr17-11782218231222339] Wakeman SE Larochelle MR Ameli O , et al. Comparative effectiveness of different treatment pathways for opioid use disorder. JAMA Netw Open. 2020;3: e1920622. 10.1001/jamanetworkopen.2019.20622 PMC11143463 32022884

[18] Walters SM Perlman DC Guarino H Mateu-Gelabert P Frank D. Lessons from the first wave of COVID-19 for improved medications for opioid use disorder (MOUD) treatment: benefits of easier access, extended take homes, and new delivery modalities. Subst Use Misuse. 2022;57:1144-1153. 35443862 10.1080/10826084.2022.2064509 PMC9709780

[19] Keyes KM Rutherford C Hamilton A , et al. What is the prevalence of and trend in opioid use disorder in the United States from 2010 to 2019? Using multiplier approaches to estimate prevalence for an unknown population size. Drug Alcohol Depend Rep. 2022;3:100052. 10.1016/j.dadr.2022.100052 PMC9248998 35783994

[20] Krawczyk N Rivera BD Jent V , et al. Has the treatment gap for opioid use disorder narrowed in the U.S.?: A yearly assessment from 2010 to 2019. Int J Drug Policy. 2022;110:103786. 10.1016/j.drugpo.2022.103786 PMC10976290 35934583

[21] Mauro PM Gutkind S Annunziato EM Samples H. Use of medication for opioid use disorder among U.S. Adolescents and adults with need for opioid treatment, 2019. JAMA Netw Open. 2022;5:e223821. 10.1001/jamanetworkopen.2022.3821 PMC8943638 35319762

[22] Apsley HB Vest N Knapp KS , et al. Non-engagement in substance use treatment among women with an unmet need for treatment: A latent class analysis on multidimensional barriers. Drug Alcohol Depend. 2023;242:109715. 10.1016/j.drugalcdep.2022.109715 PMC9847351 36495652

[23] Mancher M Leshner AI ; National Academies of Sciences, Engineering, and Medicine. Barriers to broader use of medications to treat opioid use disorder. In: National Academies of Sciences, Engineering, and Medicine, ed. Medications for Opioid Use Disorder Save Lives. National Academies Press; 2019;19-44. 30896911

[24] Dickson-Gomez J Spector A Weeks M , et al. “You’re not supposed to be on it forever”: Medications to treat opioid use disorder (MOUD) related stigma among drug treatment providers and people who use opioids. Subst Abuse Res Treat. 2022;16:11782218221103859. 10.1177/11782218221103859 PMC9243471 35783464

[bibr25-11782218231222339] Madden EF Prevedel S Light T Sulzer SH. Intervention stigma toward medications for opioid use disorder: a systematic review. Subst Use Misuse. 2021;56:2181-2201. 34538213 10.1080/10826084.2021.1975749

[26] Richard EL Schalkoff CA Piscalko HM , et al. “You are not clean until you’re not on anything”: perceptions of medication-assisted treatment in rural Appalachia. Int J Drug Policy. 2020;85:102704. 10.1016/j.drugpo.2020.102704 PMC8018539 32173274

[27] Chou JL Patton R Cooper-Sadlo S , et al. Stigma and medication for opioid use disorder (MOUD) among women. Int J Ment Health Addict. 2022;20:3262-3273.

[bibr28-11782218231222339] Fiddian-Green A Gubrium A Harrington C Evans EA. Women-reported barriers and facilitators of continued engagement with medications for opioid use disorder. Int J Environ Res Public Health. 2022;19:9346. 35954700 10.3390/ijerph19159346 PMC9368271

[29] Martin CE Almeida T Thakkar B Kimbrough T. Postpartum and addiction recovery of women in opioid use disorder treatment: A qualitative study. Subst Abuse. 2022;43:389-396. 10.1080/08897077.2021.1944954 PMC8720325 34214405

[30] Cernasev A Hohmeier KC Frederick K Jasmin H Gatwood J. A systematic literature review of patient perspectives of barriers and facilitators to access, adherence, stigma, and persistence to treatment for substance use disorder. Explor Res Clin Soc Pharm. 2021;2:100029. 10.1016/j.rcsop.2021.100029 PMC9029901 35481114

[31] Staton M Pike E Tillson M Lofwall MR. Facilitating factors and barriers for use of medications to treat opioid use disorder (MOUD) among justice-involved individuals in rural Appalachia. J Community Psychol. 2023. doi:10.1002/jcop.23029 PMC10505241 36930568

[32] Chou JL McDowell D Bennett DS , et al. Family support and medication for opioid use treatment for women: a mixed methods study. J Soc Work Pract Addict. 2022;1-15. doi:10.1080/1533256X.2022.2109271

[33] Allen EK. Justice-involved women: Narratives, marginalization, identity and community reintegration. Affilia. 2018;33:346-362.

[bibr34-11782218231222339] Bleasdale J Morse DS Cerulli C , et al. Women’s motivators to engage in opioid use disorder treatment while enrolled in an opioid intervention court. Subst Use Misuse. 2022;57:1035-1042. 35382688 10.1080/10826084.2022.2058704 PMC9215118

[35] Smith S Wickliffe J Rivera-Newberry I , et al. A systematic evaluation of barriers and facilitators to the provision of services for justice-involved women. Community Health. 2020;45:1252-1258. 10.1007/s10900-020-00894-w 32737745

[36] Krawczyk N Mojtabai R Stuart EA , et al. Opioid agonist treatment is highly protective against overdose death among a US statewide population of justice-involved adults. Am J Drug Alcohol Abuse. 2021;47:117-126.

[37] Walley AY Lodi S Li Y , et al. Association between mortality rates and medication and residential treatment after in-patient medically managed opioid withdrawal: a cohort analysis. Addiction. 2020;115:1496-1508. 32096908 10.1111/add.14964 PMC7854020

[38] Alho H Dematteis M Lembo D , et al. Opioid-related deaths in Europe: strategies for a comprehensive approach to address a major public health concern. Int J Drug Policy. 2020;76:102616. 10.1016/j.drugpo.2019.102616 31855706

[39] Kumar N Oles W Howell BA , et al. The role of social network support in treatment outcomes for medication for opioid use disorder: a systematic review. J Subst Abuse Treat. 2021;127:108367. 10.1016/j.jsat.2021.108367 PMC9022048 34134871

[40] Broman MJ Pasman E Brown S , et al. Social support is associated with reduced stigma and shame in a sample of rural and small urban adults in methadone treatment. Addict Res Theory. 2023;31:37-44.

[41] Stack E Hildebran C Leichtling G , et al. Peer recovery support services across the continuum: in community, hospital, corrections, and treatment and recovery agency settings - a narrative review. J Addict Med. 2022;16:93-100. 33560695 10.1097/ADM.0000000000000810 PMC8339174

[bibr42-11782218231222339] Tookes H Ucha J Rodriguez AE , et al. Recruitment into a clinical trial of people living with uncontrolled HIV infection who inject drugs: a site case report from the CTN 67 CHOICES Study. J Behav Health Serv Res. 2022;49: 240-251. 34590235 10.1007/s11414-021-09771-3 PMC8960468

[43] Jones AA Dyer TV Das A , et al. Risky sexual behaviors, substance use, and perceptions of risky behaviors among criminal justice involved women who trade sex. J Drug Issues. 2019;49:15-27. 33828337 10.1177/0022042618795141 PMC8022863

[44] Beckman KL Williams EC Hebert P , et al. The impact of military sexual trauma and gender on receipt of evidence-based medication treatment among veterans with opioid use disorder. J Subst Abuse Treat. 2022;139:108775. 10.1016/j.jsat.2022.108775 35317959

[45] Jones AA Gerke T Striley CW , et al. A longitudinal analysis of the substance abuse, violence, and HIV/AIDS (SAVA) syndemic among women in the criminal justice system. J Psychoactive Drugs. 2019;51:58-67. 30626264 10.1080/02791072.2018.1562132 PMC6386603

[46] Hooker SA Sherman MD Lonergan-Cullum M Nissly T Levy R. What is success in treatment for opioid use disorder? Perspectives of physicians and patients in primary care settings. J Subst Abuse Treat. 2022;141:108804. 10.1016/j.jsat.2022.108804 35643586

[47] Techau A Gamm E Roberts M Garcia L. The lived experience of medication for opioid use disorder: qualitative metasynthesis. J Addict Nurs. 2023;34: E119-E134. 10.1097/JAN.0000000000000475 PMC10510791 37669351

[48] Tillson M Fallin-Bennett A Staton M. Providing peer navigation services to women with a history of opioid misuse pre- and post-release from jail: a program description. Clin Transl Sci. 2022;6:e106. 10.1017/cts.2022.441 PMC9453575 36128341

[49] Reed MK Smith KR Ciocco F , et al. Sorting through life: evaluating patient-important measures of success in a medication for opioid use disorder (MOUD) treatment program. Subst Abuse Treat Prev Policy. 2023;18:4. 36641478 10.1186/s13011-022-00510-1 PMC9839958

