# Supplementary material for: Women and Treatment for Opioid Use Disorder: Contributors to Treatment Success From the Perspectives of Women in Recovery, Women With Past Attempts in Drug Treatment, and Health and Criminal Justice Professionals
Source: Subst Use. 2024 Jan 10;18:11782218231222339. doi: 10.1177/11782218231222339 (PMC10906498; doi:10.1177/11782218231222339)
Supplement: sj-docx-1-sat-10.1177_11782218231222339 – Supplemental material for Women and Treatment for Opioid Use Disorder: Contributors to Treatment Success From the Perspectives of Women in Recovery, Women With Past Attempts in Drug Treatment, and Health and Criminal Justice Professionals [file sj-docx-1-sat-10.1177_11782218231222339.docx]

***Questions for Qualitative Study: Women***

*This is a semi-structured questionnaire; however, it allows participants to introduce as much unique, historical, and contextual detail as possible based on their experiences with drug use, drug treatment, and the criminal justice system.*

*Interviewers should listen for themes related to drug use, drug treatment, and the judicial system during the interview and probe accordingly.*

*Common probes:*

- *What do you mean by…?*
- *How did that make you feel?*
- *What did you think about that?*
- *How was that for you?*
- *Explain what you mean.*
- *Tell me more about that.*
- *Use of silence, mhm, uh-huh to encourage further elaboration.*
- *Can you give an example?*

**Section 1: *The interviewer will discuss the purpose of the interview***

I’d like to ask you some questions about your life and your experiences with opioid use, drug treatment programs like methadone or suboxone treatment, and your thoughts about ways to improve drug treatment for women. Please don’t answer any questions that you don’t want to. What I learn from you today will hopefully be used to improve drug treatment programs. Everything we discuss today will be kept confidential. There will be nothing to identify you personally, and we will only share what you say here today with the other research team members. The interview will take one hour to complete. You will be sent a $50 gift card after the interview in appreciation of your time.

Do you have any questions before we start?

I may take notes during our conversation to help me remember things.

Can I turn on the recorder?

**Section 2: *Background Information and Personal History***

1. Age, race/ethnicity/gender identity/LQBTQ identity
2. Highest grade completed in school
3. Tell us about yourself—how do you describe yourself and anything you would want us to know about you.

**Section 3: *Support Services***

1. What social services do you currently use (job assistance, food stamps, WIC, etc.)?
   1. What services would you like to receive that you are not currently receiving?

**Section 4: *Drug Treatment Experiences***

1. Tell me about the last time have tried to decrease your drug use or quit using drugs on your own.
2. Tell me about any times when you have entered a drug treatment program.

*Use the following probes* ***for each program and each session in treatment****: Probe for:*

- *What kind? Voluntary “cold turkey” detox, forced detox, substitution treatment, a support group like Alcoholics Anonymous.*
- *How did you learn about each program?*
- *How easy or difficult was each program to access?*
- *Reasons they entered treatment*
- *Reasons they left treatment*
- *How long they were in treatment each time*
- *If they had adherence issues and if so, why experiences during treatment*
- *How relationships with treatment providers, staff, and counselors influenced their treatment experience*
- *Interactions between drug treatment and criminal justice system*

1. When you reflect on your time in drug treatment, what are the first things or experiences that come to your mind?
2. What was the most helpful when you were in drug treatment? What helps women stay in drug treatment?
3. What was the least helpful when you were in drug treatment? What do you think is the main reason women drop out of drug treatment?
4. When you started drug treatment *(****ask for each time in treatment)****,* did you have any goals?

- If so, what were they?
- Were your goals the same goals as the treatment center had for you?

1. Tell me about times when you felt mistreated (e.g., demeaning language, concerns not taken seriously, not given medications) by staff because you:
   - Are a woman
   - Use drugs
   - What did you do in these situations? (e.g., go somewhere else, not return for follow-up, complain to management)

**Section 5: *Competing Demands***

1. Income: How do you make ends meet?

*Probe for: tax paying job, last employment, gvt assistance, support from friends/ family, illicit employment*

- How does that affect you getting into drug treatment?

1. Where are you currently staying at night?

- Any history of homelessness?
- How does that affect you getting into drug treatment?

1. Transportation: How do you get around- do you have a car, walk, take the bus?

- How long does it take you to get to your drug treatment appointments?
- How do you pay for this?

1. Childcare: (*if indicated children live with them*) Who cares for your kids while you’re at drug treatment?

**Section 6: *Criminal Justice Involvement***

1. How many times have you been incarcerated or on probation or parole?
2. Did you use drugs before you were incarcerated (*ask for each time)?*
   1. What types of drugs
3. Did you use drugs when you were released?

- Walk me through what happened- how soon after you were released did you start using drugs?
- How did that affect your probation/ parole?

1. Did you start drug treatment while incarcerated or when you were released?

- If you started while you were incarcerated, did you have trouble staying in treatment when you were released?
- Why or why not?

1. How did criminal justice involvement impact your life after you were off of probation or parole?

- Did it change the way you used drugs?
- Was your life different before and after?

1. What worries you most about your life?
2. What do you wish the drug treatment health workers, court system and politicians knew about your life?

**Section 7:**

***Overdose***

1. There are a lot of drug overdoses happening in the US right now. Is there anything that can be done to slow down overdose deaths that you think is not being done enough or at all?
2. What do you do to avoid drug overdoses?

- Has anyone ever given you any advice on how to avoid an overdose?
- What do you see other women who use drugs do to avoid drug overdoses?

***Suggestions for Drug Treatment Programs***

1. If you had all the money and resources in the world to design the perfect drug treatment program for women, what kind of drug treatment program would you create?

- How would this drug treatment program change if the women were on parole or probation?
- What type of services do you think should be included?

1. Do you think there are issues specific to women that drug treatment for opioid use did not address at all or well?
2. Is there anything we should know about women, drug treatment, criminal justice involvement, and any related issue that you think is important that we did not already ask about?

*End interview by thanking participants and asking if there is anything else that they would like to say or that they think we should know.*

***Questions for Qualitative Study: MOUD Providers***

*This is a semi-structured questionnaire, however, allow participants to introduce as much unique, historical, and contextual detail as possible based on their experiences with drug use, drug treatment, and the criminal justice system.*

*Interviewers should be conscious of listening for themes related to drug use, drug treatment, and the judicial system during the interview, and probe accordingly.*

*Common probes:*

- *What do you mean by…?*
- *How did that make you feel?*
- *What did you think about that?*
- *How was that for you?*
- *Explain what you mean.*
- *Tell me more about that.*
- *Use of silence, mhm, uh-huh to encourage further elaboration.*
- *Can you give an example?*

**Section 1: *The interviewer will discuss the purpose of the interview***

I’d like to ask you some questions about your life, and your experiences with treating women who use opioids and other drugs, drug treatment programs like methadone or suboxone treatment, and your thoughts about ways to improve drug treatment for women. Please don’t answer any question that you don’t want to. What I learn from you today will hopefully be used to improve drug treatment programs. Everything we discuss today will be kept confidential. There will be nothing to personally identify you, and we will only share what you say here today with the other research team members. The interview will take one hour to complete. In appreciation of your time, you will be sent a $50 gift card after the interview.

Do you have any questions before we start?

I may take notes during our conversation to help me remember things.

Can I turn on the recorder?

**Section 2: *Background Information and Personal and Employment History***

1. Age, race/ethnicity/gender identity/LGBTQ identity
2. Brief educational and employment history
3. Length of time working in addiction medicine and with women who use drugs

**Section 3: Support Services and Background of Patients**

1. What services do you currently offer to your female patients with substance use disorders?
2. What are the demographics of the women with substance use disorders you treat? (race/ethnicity, age, children, marital status, housing status, social support)
3. What are the most common substances used and common routes of administration of drugs by the women you treat?
   1. Have you noticed changes in the types of drugs used and the way the drugs are used in the last 5 years?

**Section 4: Thoughts on Support Services for Women who Use Drugs**

1. Reflect on your time helping women in drug treatment. Are there one or two lessons you learned that changed or challenged your viewpoints on how to best help women with substance use disorders?
   1. What were they, and how did they change or challenge your viewpoints?
2. Do you think there are issues specific to women that drug treatment programs for opioid use disorder and other drugs do not address at all or well?
3. What have you noticed to be the most helpful to women in drug treatment for opioid use and other substances?

**Section 5: Barriers to Successful Treatment for Women who Use Drugs**

1. What do you consider as being successfully treated for substance use disorder?
   1. What does that look like for women who use drugs?
2. What have you noticed to be the least helpful to women in drug treatment for opioid use and other substances? What do you think is the main reason women drop out of drug treatment?
3. Are treatment costs, housing instability, transportation, disability, or childcare barriers to drug treatment for women in your program?
   1. If so, can you give some notable examples?
4. What worries you most about the drug treatment programs available to women currently?
   1. What do you think drug treatment programs, especially programs that offer medications for opioid use disorder, need the most help with?
5. From your viewpoint, what are the biggest challenges in the women's lives in your drug treatment program?
   1. What do you think can best help women and the challenges you mentioned?
6. What strategies have you used or would you suggest to decrease the number of women who drop out of treatment?
7. When you think about the women you consider successfully treated and women who drop out of treatment, what are the differences?

**Section 6: Criminal Justice**

1. Have you observed any differences between women in drug treatment who were involved in criminal justice in any way (recently released from incarceration, on parole, or probation) and women in drug treatment who were not involved in the justice system in any way? If so, what were the differences?
2. What do you wish the court system knew about women's lives in drug treatment and also involved in the criminal justice system?

**Section 7: Overdose**

1. There are a lot of drug overdoses happening in the US right now. Is there anything that can be done to slow down overdose deaths that you think is not being done enough or at all?
2. What methods have you heard women use to avoid drug overdoses?
   1. What are the most common methods?
   2. What are unique ones?

**Section 8: Optimal Ways to Improve Outcomes**

1. If you had all the money and resources in the world to design the perfect drug treatment program for women, what kind of drug treatment program would you create?
   1. How would this drug treatment program change if the women were on parole or probation?
2. What do you think could be done to make drug treatment programs more accessible to women?
   1. What do you think could make them more accessible to women involved in the criminal justice system?
3. What do you wish politicians and researchers knew about treating women with opioid use disorder---and substance use disorders in general--that could improve drug treatment outcomes for women?
4. Is there anything we should know about women, drug treatment, criminal justice involvement, and any related issue that you think is important that we did not already ask about?

*End interview by thanking participants and asking if there is anything else they would like to say or think we should know.*

***Questions for Qualitative Study: Criminal Justice Professionals***

*This is a semi-structured questionnaire, however, allow participants to introduce as much unique, historical, and contextual detail as possible based on their experiences with drug use, drug treatment, and the criminal justice system.*

*Interviewers should listen for themes related to drug use, drug treatment, and the judicial system during the interview and probe accordingly.*

*Common probes:*

- *What do you mean by…?*
- *How did that make you feel?*
- *What did you think about that?*
- *How was that for you?*
- *Explain what you mean.*
- *Tell me more about that.*
- *Use of silence, mhm, uh-huh to encourage further elaboration.*
- *Can you give an example?*

**Section 1: *The interviewer will discuss the purpose of the interview***

I want to ask you some questions about your life, your experiences with the criminal justice system, and with women who use opioids and other drugs and are incarcerated, and your thoughts about improving drug treatment for women. Please don’t answer any questions that you don’t want to. What I learn from you today will hopefully be used to improve drug treatment programs and related services for women who use drug and are involved in the criminal justice system. Everything we discuss today will be kept confidential. There will be nothing to identify you personally, and we will only share what you say here today with the other research team members. The interview will take one hour to complete. You will be sent a $50 gift card after the interview in appreciation of your time.

Do you have any questions before we start?

I may take notes during our conversation to help me remember things.

Can I turn on the recorder?

**Section 2: *Background Information and Personal History***

1. Age, race/ethnicity/gender identity/LGBTQ identity
2. Brief educational and employment history
3. Length of time working in criminal justice and with women in the criminal justice system

**Section 3: Overview of Women Who Use Drugs and the Criminal Justice System**

1. When a woman with drug addiction issues comes into your jail/prison facility, what happens to them?
   1. Do they get help with withdrawal symptoms at all? If so, what support/services do they receive?
   2. Are there drug treatment programs for women in your facility? If so, do you think the drug treatment is helpful to the women?
2. Have you heard women in your facility with issues with drug addiction talk about the challenges they face in the facilities you have worked in?
   1. If so, what are some of the comments?
3. Reflect on your time working with women involved in the justice system. Are there one or two lessons you learned that changed or challenged your views on how to best help women with issues with drug addiction?
   1. What were they, and how did they change or challenge your viewpoints?
4. From your viewpoint, what are the biggest challenges in the lives of the women who use drugs and are involved in the criminal justice system?
   1. What do you think can best help women and the challenges you mentioned?
5. What concerns you most about the criminal justice system’s methods of dealing with women who use drugs?
   1. What do you think the criminal justice system needs to change to better help women who use drugs?
   2. What do you wish the court system knew about the day-to-day lives of women who use drugs and are also involved in the criminal justice system?
6. Have you observed any differences between women involved in criminal justice in any way (recently released from incarceration, on parole, or probation) who used drugs and women involved in the justice system who did not use drugs?
   1. If so, what were the differences?

**Section 4: Thoughts on Services Provided for Women Who Use Drugs in the Criminal Justice System**

1. Do you think there are issues specific to women who are incarcerated, on parole, or probation and use opioids and/or other drugs that the justice system does not address at all or well?
2. What have you noticed to be the most helpful to women involved in the justice system who use drugs?
3. What have you noticed to be the least helpful to women involved in the justice system who use drugs?
4. Have you noticed any differences between women who use drugs and are repeatedly in the criminal justice system and women who use drugs but do not return to the criminal justice system once they are released?
   1. If so, what are the differences?

**Section 5: Optimal Ways to Help Women Who Use Drugs in the Criminal Justice System**

1. If you had all the money and resources in the world to design the perfect drug treatment program for women—particularly women who are on parole or probation-- what kind of drug treatment program would you create?
2. What do you wish politicians and researchers knew about incarcerated women or women on parole or probation who use drugs?
3. There are a lot of drug overdoses happening in the US right now. Is there anything that can be done to slow down overdose deaths that you think is not being done enough or at all?
4. Is there anything we should know about women, drug treatment, criminal justice involvement, and any related issue that you think is important that we did not already ask about?

*End interview by thanking participants and asking if there is anything else they would like to say or think we should know.*
